# The nuts and bolts of pediatric cardiac care for the economically challenged

**DOI:** 10.4103/0974-2069.52807

**Published:** 2009

**Authors:** R Krishna Kumar

**Affiliations:** Department of Pediatric Cardiology, Amrita Institute of Medical Sciences and Research Center, Cochin, Kerala, India

A large number of people live in countries that are experiencing rapid economic transition. India and China along with many other countries in Asia are now classified as emerging economies. If current economic growth rates are sustained, the center of gravity of the world's economy is expected to shift to Asia by 2050.[1] However, human development in most of these countries has not matched economic development. The disparity between economic prosperity and most parameters that determine human development, such as infant and under-five mortality, literacy rate and life expectancy, is particularly striking in India.[2] This is largely the result of severely dysfunctional public health systems in the government sector and a completely unregulated private sector. This allows for a chaotic health infrastructure that is substantially divorced from national priorities and results in extraordinary contrasts. Most urban metros in India have become microcosms of the globalized world and showcase bizarre contradictions. Advanced high-tech health care that caters to overseas patients (medical tourism) and a relatively small (but growing) proportion of affluent Indians is available at a short distance from urban slums where a large number of people do not have access to basic health care.

Comprehensive pediatric heart care is very resource intensive and requires a sophisticated infrastructure. It is challenging to develop a successful and effective program in the government sector. As a result, most pediatric heart programs in India are in the private sector and most procedures are expensive and largely out of reach of the average Indian.[3] The authors of the review (Cardiac care in the economically challenged: What are the options) have made an earnest effort to identify a number of potential sources (government as well as non-government organizations).[4] The information provided is of practical value and many readers of the journal will find this useful.

The authors begin the review by touching upon the relevance of pediatric cardiac care from a public health perspective. They present an emotional defense to justify why pediatric heart programs are relevant in India when there are many other readily preventable causes of childhood mortality. In this context, it is important to understand that substantial reductions in infant and under-five mortalities to levels comparable to many developed nations are achievable without any facility for infant heart surgery. Cuba and Sri Lanka are examples of two countries that have achieved very low infant and under-five mortality without establishing a center with infant heart surgery.[5] When viewed purely from a public health perspective, government subsidies for pediatric cardiac programs would amount to seriously misplaced priorities in the face of readily preventable conditions. However, it is difficult to precisely regulate health care delivery in many situations. This is especially true for democratic nations. Perhaps as a result of this situation we do have a handful of institutions in India that have established good standards of pediatric heart care with rapidly increasing numbers. A growing number of new institutions are now looking to establish comprehensive pediatric cardiac services. The total number of infant open heart operations in India has almost doubled over the last 5 years.

What are the most compelling factors that justify (or encourage) the establishment of centers with comprehensive and dedicated pediatric cardiac care in India?

- In absolute terms, there is a large disease burden simply because there is a large population of children with heart disease. With the rapid growth of the urban middle class, there are a number of families that can now afford pediatric heart care. The reduction in family size has resulted in greater willingness to allocate substantial resources for treatment of a child with heart disease.
- Many congenital heart conditions can be corrected early in infancy as “one-time fixes” with a near normal future. A single (although expensive) investment apparently translates into a lifelong benefit.
- Successful and safe infant heart surgery is often viewed as an important benchmark in quality for a number of hospital systems. A number of core hospital services will need to function at a very high level in order to make infant heart surgery safe. The spin offs from improved infrastructure and health systems in a hospital are substantial and can contribute to an overall improvement in the general level of care in a hospital.
- Health care is now developing as an industry. Apart from the growing middle class, there are opportunities for income generation through medical tourism for the developing world.
- There is a large pool of trained manpower available in India. A number of medical schools offer an excellent background in pediatrics and many pediatricians are looking to specialize. Pediatric cardiology is a new and exciting specialty that offers a great deal of intellectual challenge. Similarly, the human resources for paramedical, nursing and technical services are abundant and they can be readily trained through structured programs.
- Recognizing the economic transition that is happening in India, there is likely to be an enormous need for pediatric heart programs. If the average child in India were to have access to comprehensive heart care, we would need at least 200 large programs with an annual case load of 800-1000 open heart operations. Recognizing this long-term perspective, it may be justifiable to develop core expertise in selected large institutions and establish facilities for training manpower for the future.

While recognizing the seemingly limitless opportunity, it is also important to understand that today there are a number of barriers to effective pediatric heart care in India. They include poor recognition and management of heart disease at the level of primary care facilities, district and medical college hospitals, logistic challenges resulting from concentration of pediatric heart centers in selected cities and cost of care. Dedicated pediatric cardiac surgeons are in short supply and little is being done to address the problem. There are no formal training programs for pediatric cardiac surgery and this will become a major bottleneck as new centers are established.

These barriers need to be overcome. A number of initiatives have attempted to address some of these barriers. The pediatric cardiac society of India has been working on educating pediatricians and neonatologists on recognition of heart disease. Formal fellowship training programs have been established in pediatric cardiology. Many cost-effective strategies have been developed for catheter interventions and congenital heart surgery in Indian centers. Realistic guidelines for management of common congenital heart lesions are being developed.

A number of challenges lie ahead. The most fundamental challenge however is in making comprehensive pediatric heart care available to the average child in the region. Obtaining a list of potential donors and insurance providers is a reasonable start. But, core issues will need to be addressed before government and non-government subsidies can be offered as a realistic solution for the average child in the country:

1. How sustainable are many of the current insurance and donor programs? Will the governments continue to find resources to subsidize pediatric heart care for the years to come? How can they justify this expense from a public health perspective?
2. Bottlenecks in the form of capacity to deliver care: Most pediatric heart programs in India are overloaded and have significant waiting lists.
3. Potential for exploitation by those who can afford: Most systems that deliver subsidized care are flawed, in that most subsidies do not reach those who truly deserve them.
4. Potential for inappropriate use by hospitals that cater to patients obtaining government subsidies through unwarranted procedures.
5. The ethical dilemma of prioritizing pediatric heart care in India. Specific ethical questions include: Should multistaged palliations for single-ventricle physiology be performed? Should hypoplastic left heart syndrome be treated? Should we develop pediatric cardiac transplantation in selected centers? Etc.

## CONCLUSIONS

There are perhaps enough reasons to justify the presence of pediatric heart care in India as we make the transition to the future. A number of issues will need to be deliberated upon and implemented as a national consensus. Practice guidelines recognizing our resource limitations will need to be developed. These guidelines will serve as useful references for insurance providers. Regulations and audits on how insurance and government subsidy is distributed are urgently needed to ensure equity. Simultaneously, a number of basic initiatives to reduce costs of care should be pursued. These include training manpower locally, continued efforts toward cost containment through appropriate treatment strategies and development of indigenous technology.
